# Cambrian stem-group annelids and a metameric origin of the annelid head

**DOI:** 10.1098/rsbl.2015.0763

**Published:** 2015-10

**Authors:** Luke Parry, Jakob Vinther, Gregory D. Edgecombe

**Affiliations:** 1 Bristol Life Sciences Building, University of Bristol, 24 Tyndall Avenue, Bristol BS8 1TH, UK; 2 Department of Earth Sciences, Natural History Museum, Cromwell Road, London SW7 5BD, UK

**Keywords:** Annelida, *Canadia*, palps, Cambrian

## Abstract

The oldest fossil annelids come from the Early Cambrian Sirius Passet and Guanshan biotas and Middle Cambrian Burgess Shale. While these are among the best preserved polychaete fossils, their relationship to living taxa is contentious, having been interpreted either as members of extant clades or as a grade outside the crown group. New morphological observations from five Cambrian species include the oldest polychaete with head appendages, a new specimen of *Pygocirrus* from Sirius Passet, and an undescribed form from the Burgess Shale. We propose that the palps of *Canadia* are on an anterior segment bearing neuropodia and that the head of *Phragmochaeta* is formed of a segment bearing biramous parapodia and chaetae. The unusual anatomy of these taxa suggests that the head is not differentiated into a prostomium and peristomium, that palps are derived from a modified parapodium and that the annelid head was originally a parapodium-bearing segment. *Canadia*, *Phragmochaeta* and the Marble Canyon annelid share the presence of protective notochaetae, interpreted as a primitive character state subsequently lost in *Pygocirrus* and *Burgessochaeta*, in which the head is clearly differentiated from the trunk.

## 1. Introduction

The annelid fossil record reveals morphological disparity in extinct groups of polychaetes, especially in the Palaeozoic. This includes higher taxonomic diversity in Palaeozoic versus extant eunicidans [1] and identification of machaeridians as polychaetes with unique calcitic armour [2]. Although annelid fossils are rare, they provide unique character combinations and body plans.

There are currently eight polychaete species known from carbonaceous compressions in Burgess Shale-type Lagerstätten. The oldest among these are from the Early Cambrian Sirius Passet [3] and Guanshan biotas [4], with younger fossils from the Burgess Shale [5,6]. These fossils share no derived characters with any extant clades and are currently interpreted as stem-group annelids [3,6,7]. While *Canadia* was previously interpreted as a member of the Phyllodocida [8], the absence of jaws, antennae and parapodial cirri argues for a placement outside this group and, crucially, the absence of pygidial cirri suggests that both *Canadia* and *Burgessochaeta* are stem-group annelids [6]. Compared to extant annelids, these taxa are morphologically simple, possessing characters such as homonomous segmentation and well-developed biramous parapodia with simple chaetae but lacking aciculae. Some taxa bear a single pair of anterior appendages, including *Burgessochaeta*, *Canadia* and *Peronochaeta* from the Burgess Shale [5]. Head appendages are also described from the single-known specimen of *Guanshanchaeta* [4], are absent in *Phragmochaeta* and were previously uncertain in the incomplete *Pygocirrus* [3]. Morphological evidence indicates that these contractile appendages are palps [6]. Anterior regions of *Insolicorypha* and *Stephenoscolex* are currently unknown [6] and palps are only putatively present in a single specimen of *Peronochaeta* [5]. Consequently, these taxa are not considered herein.

Palps are unique head appendages of annelids. They are used either in feeding or as sensory structures, showing a diversity of external morphology, attachment and position on the head. Palps originate from either the peristomium or prostomium and are either smooth and sensory, possess a longitudinal ciliated groove to transport food particles, or have adhesive papillae [9]. Palps are typically paired, but are elaborated into a feeding crown in a clade of Fabriciidae, Sabellidae and Serpulidae [10]. Despite this diversity, polychaete palps share a common pattern of innervation, and hence are considered homologous [11]. Palps have long been considered a phylogenetically significant character, either uniting a major clade of polychaetes [12] or a synapomorphy of Annelida that underwent reduction and loss numerous times [6,7,13].

Herein we describe new anatomical observations of palps from Cambrian taxa, clarifying their attachment and distribution. This includes new material from Sirius Passet, notably a specimen of *Pygocirrus* with head appendages. We present evidence that the palps of *Canadia* are attached to a parapodium-bearing segment anterior to the mouth and discuss the implications of these findings in the context of annelid head evolution.

## 2. Observations

### (a) Burgess Shale

*Canadia spinosa* possesses posteriorly directed flattened notopodial paleae, a single pair of anterior palps and interramal parapodial gills that resemble the ctenidia of some molluscs [5]. Like *Phragmochaeta*, the paleae are posteriorly directed, performing a presumed protective function.

**Figure 1.**
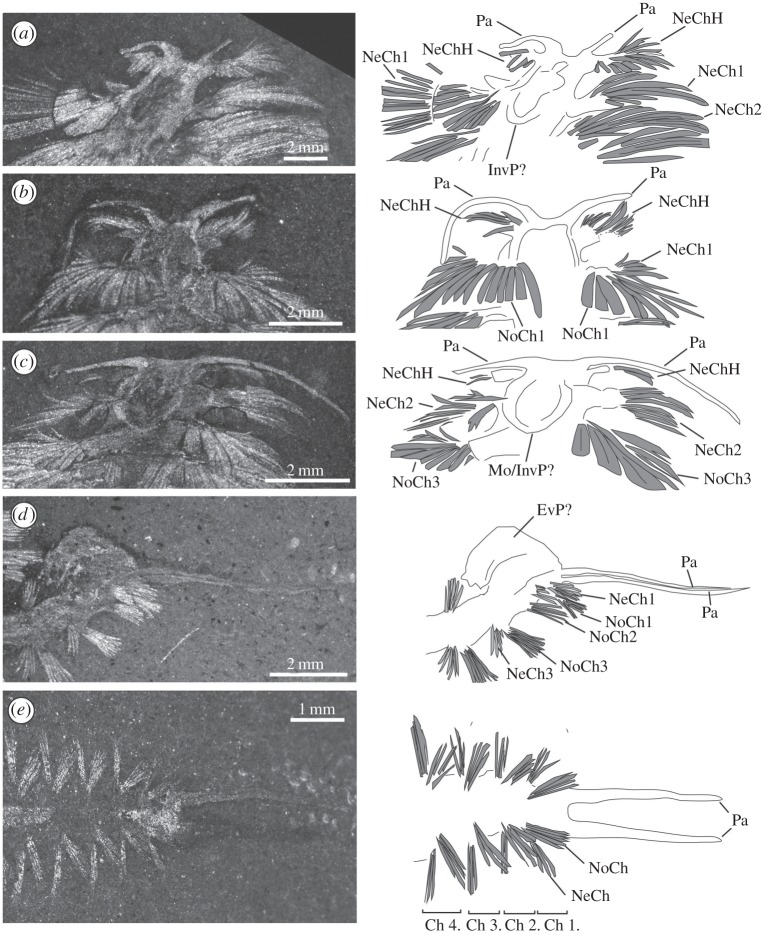
Burgess Shale polychaetes. (*a*) *Canadia spinosa*, United States National Museum of Natural History (USNM) 199655. (*b*) *Canadia spinosa* USNM83929b. (*c*) *Canadia spinosa* USNM275517. (*d*) *Burgessochaeta setigera* USNM198701. (*e*) *Burgessochaeta setigera* USNM198699. Pa, palps; InvP, inverted proboscis; Mo, mouth; EvP, everted proboscis; NoCh, notochaetae; NeCh, neurochaetae. Numbering indicates segmental identity, H identifies chaetae on the head.

We show here that the palps of *Canadia*, unlike those in living annelids, are attached to a neuropodium-bearing first segment. Despite variable orientations, the palps are consistently dorsal of the neuropodia of this segment (figure 1*a*–*c*), effectively in the place of the notopodium (figure 1*a*). While Conway Morris [5] figured a structure anterior of the palps in USNM19730 and 83929B, we consider these structures to be dubious. Such a structure was not observed in any other specimen, an accompanying dark stain indicates that the body wall has ruptured in USNM19730 (electronic supplementary material, figure S1d), and it is barely visible in USNM83929b (figure 1*b*).

In two specimens figured here, a large, rounded structure occurs ventrally between the palp-bearing chaetiger and the second chaetiger (figure 1*c*). This structure represents either the outline of the mouth or a partly everted pharynx flattened beneath the specimen during burial. This suggests that the mouth of *Canadia* was located between the first palp-bearing chaetiger and the second biramous chaetiger.

*Burgessochaeta setigera* is characterized by equant parapodial rami, paired palps and simple chaetae with bifid tips [5]. Uniramous parapodia on the anteriormost segment have previously been described [5] but the identified structures are likely the tips of the notochaetae of the first chaetiger in USNM198699 (figure 1*e*), and biramous parapodia are visible on the first segment of other specimens originally figured as uniramous by Conway Morris [5] (electronic supplementary material, figure 1*a*,*b*). Consequently, we observe biramous parapodia on all segments. The palps are clearly differentiated from the body segments, are not in close association with parapodia as in *Canadia* and are directed anteriorly where present.

**Figure 2.**
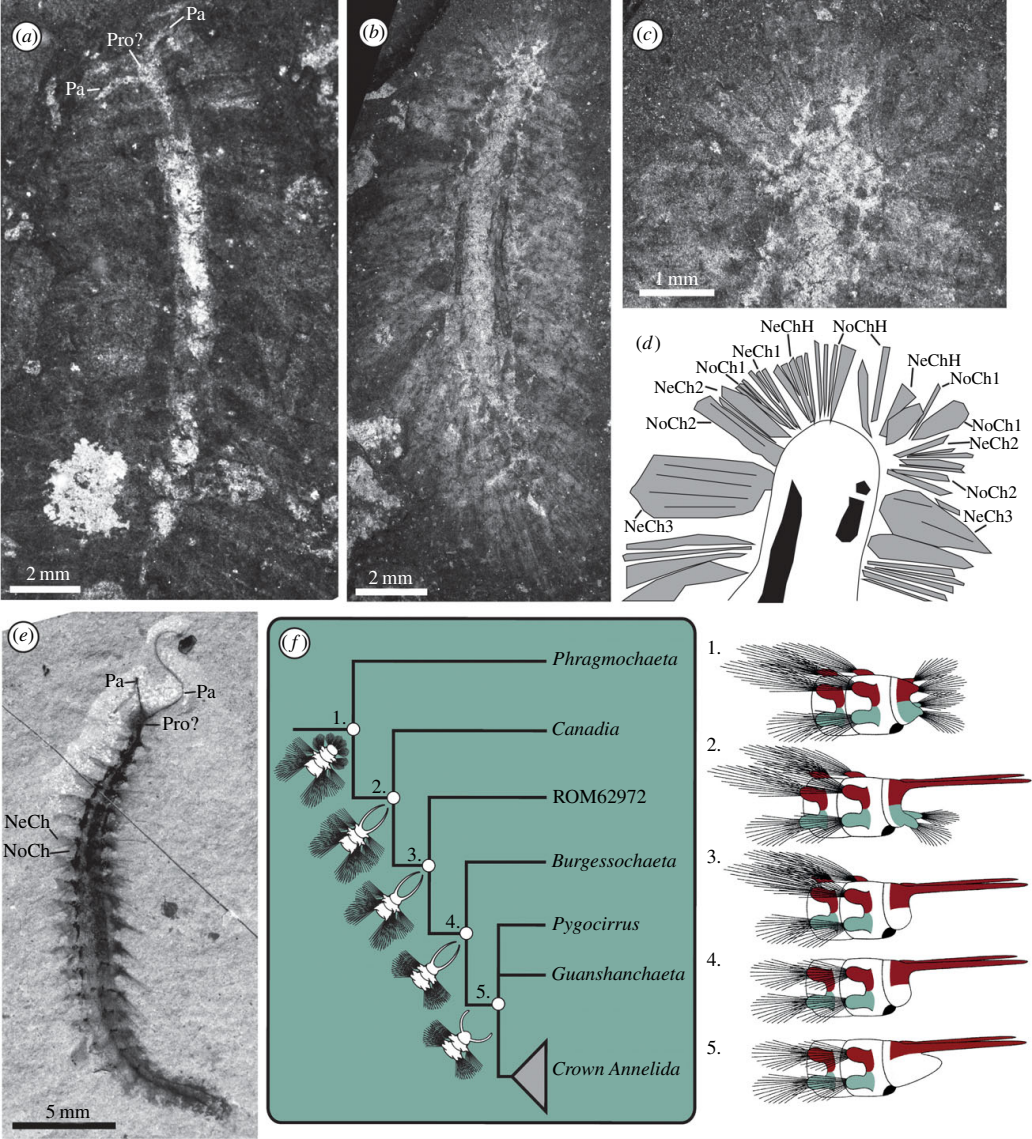
(*a*) *Pygocirrus butyricampum*, Geological Museum of Copenhagen (MGUH), with palps. (*b*) *Phragmochaeta canicularis*, MGUH3088, showing anterior chaetae surrounding the head. (*c*) Anterior region of (*b*). (*d*) Interpretive drawing of (*c*) showing the position of anterior chaetal bundles; labelling as per figure 1. (*e*) ROM62927, undescribed Marble Canyon polychaete. (*f*) Hypothetical cladogram of Cambrian taxa, drawings at right indicate the morphology of ancestors at numbered nodes. Colour indicates segmental homologues.

A Burgess Shale biota from Marble Canyon in the Canadian Rockies includes a new species initially compared with *Burgessochaeta* [14]. This taxon presents a combination of anteriorly directed palps situated on a structure differentiated from the trunk and a dorsal covering of protective notochaetae (figure 2*e*; electronic supplementary material, figure S2*a*,*b*)*.* The morphology of the notopodia is similar to *Phragmochaeta*, emerging from a notopodial lobe rather than a dorsal ridge (electronic supplementary material, figure S2*b*).

### (b) Sirius Passet

*Pygocirrus butyricampum* was previously known only from two incomplete specimens including a posterior fragment [3] with a single pair of pygidial cirri and biramous parapodia with similar rami. New material shows a single pair of palps (figure 2*a*). These are known from a single specimen and the precise attachment is unclear (i.e. whether they are prostomial or peristomial). Unlike the other Cambrian taxa with palps, a lobe lies anterior of the palps, possibly representing the prostomium (figure 2*a*; electronic supplementary material, figure S2*c*). The two rami of the parapodia are approximately equant, a rare condition in polychaetes as a whole but shared with *Burgessochaeta*.

*Phragmochaeta* possesses posteriorly directed notochaetae that form a dorsal ‘thatch’ [15]. The relative length and arrangement of the neuro- and notochaetae vary along the body. Anterior notochaetae are more laterally directed and approximately equal to neurochaetae in length, whereas posterior notochaetae are highly elongated and posteriorly directed (figure 2*b*). Unlike the dorsal paleae of *Canadia*, attached to notopodial ridges, the notochaetae of *Phragmochaeta* are situated on parapodial lobes.

The anterior region of *Phragmochaeta* terminates as a single segment with biramous parapodia, but lacks anterior structures identifiable as the peristomium, prostomium or paired palps [15] (figure 2*c*,*b*). An everted pharynx has not been observed, although this character is rarely preserved. Aciculae, jaws and parapodial cirri are likewise absent.

## 3. Discussion

Our observations suggest that posteriorly directed protective notochaetae are a widespread character among Cambrian annelids, being present in *Phragmochaeta*, *Canadia* and ROM62972. Protective notochaetae were considered a likely plesiomorphic character by Westheide [16], who hypothesized that annelids evolved from an epibenthic ancestor with a dorsal covering of notochaetae. This hypothesis was influenced by the interpretation of *Wiwaxia* as closely related to annelids, but *Wiwaxia* is now considered a total-group mollusc [17]. Dorsal protective chaetae have not featured in recent discussions of the annelid ancestor based on phylogenomic studies [18] but re-emerge as a primitive character in our scenario, based on their presence in the most primitive members of the stem group.

The heads of the Cambrian taxa are poorly differentiated from the trunk, with parapodia occurring on the anteriormost structures in *Canadia* and *Phragmochaeta.* In extant annelids, the head is considered presegmental and consists of the oral region, peristomium and prostomium, with palps developing either in front of or behind the prototroch [19]. In *Canadia*, the structure that bears palps also posseses neuropodia and occurs pre-orally (figure 1*c*), suggesting a position comparable to the presegmental region of extant taxa. We therefore suggest that the anterior parapodium-bearing region of *Canadia* and *Phragmochaeta* is homologous with the head (prostomium) of extant annelids and that these parapodia are lost in taxa closer to the crown node, such as *Burgessochaeta*, *Pygocirrus* and ROM62972. In these taxa, the head is clearly differentiated from the body and lacks parapodia. In *Burgessochaeta* and *Pygocirrus*, protective chaetae are absent (an inferred loss), with the parapodia roughly equant and laterally directed. The presence of pygidial cirri in *Pygocirrus* places this taxon crownward of the other Cambrian taxa [3].

The morphology of ROM62972 is intermediate between the more primitive and more derived forms, possessing protective notochaetae but also a differentiated head, implying that loss of parapodia on the proto-prostomium/peristomium preceded reorganization of the notopodia so that they no longer project posteriorly. *Guanshanchaeta* has no evidence for chaetae on the head, has laterally directed notopodia and a bifid pygidium [4] and is therefore of a similar phylogenetic grade to *Pygocirrus*. This hypothesis is presented in figure 2*f*, showing the transformation from a *Phragmochaeta-*like organism with its dorsum covered in protective chaetae to a morphology like *Pygocirrus* and *Guanshanchaeta.* In this scenario, protective notochaetae were lost in the annelid stem group, with the protective chaetae of extant families, e.g. Chrysopetalidae, evolving independently.

The palps of *Canadia* and *Burgessochaeta* and anterior chaetae of *Phragmochaeta* have been interpreted as sensory rather than feeding structures [6,7]. We propose that the sensory notopodium of a *Phragmochaeta*-like animal lost chaetae and became elaborated, resulting in the condition seen in *Canadia.* The derivation of a sensory palp from a parapodium is in some ways comparable to the notopodia of Eunicida, in which the external notochaetae and parapodial lobe have been reduced, leaving only a dorsal sensory cirrus [9]. While *Owenia* and *Magelona* have recently been suggested to be early branching annelid taxa based on phylogenomic data [18], it is unclear what contribution these taxa have to understanding the origin of the annelid anterior. The head structures in this potential clade are heterogeneous, with papillated peristomial palps in *Magelona* versus grooved prostomial palps in *Owenia* [9] and consequently one or both are highly autapomorphic.

## 4. Conclusion

We present new anatomical information from Cambrian polychaetes that reinforces the interpretation of these taxa as stem-group annelids. Crucially, the head structures observed in *Canadia* and *Phragmochaeta* bear parapodia and chaetae. We present a hypothesis in which the annelid prostomium was originally limb-bearing, with palps being derived from the notopodia. We furthermore infer that protective notochaetae are primitive for total-group Annelida. This scenario proposes that the annelid head has an ancient limb-bearing origin and was also segment like, as proposed for the pygidium [20]. In this light, the annelid Bauplan involved a homonomous limb-bearing morphology extending from the prostomium along the trunk to the pygidium

## Supplementary Material

Additional images of Burgess Shale polychaetes

## Supplementary Material

Additional images of Marble Canyon and Sirius Passet polychaetes
