# Supplementary figures and images for: Cambrian stem-group annelids and a metameric origin of the annelid head
Source: Biol Lett. 2015 Oct;11(10):20150763. doi: 10.1098/rsbl.2015.0763 (PMC4650189; doi:10.1098/rsbl.2015.0763)

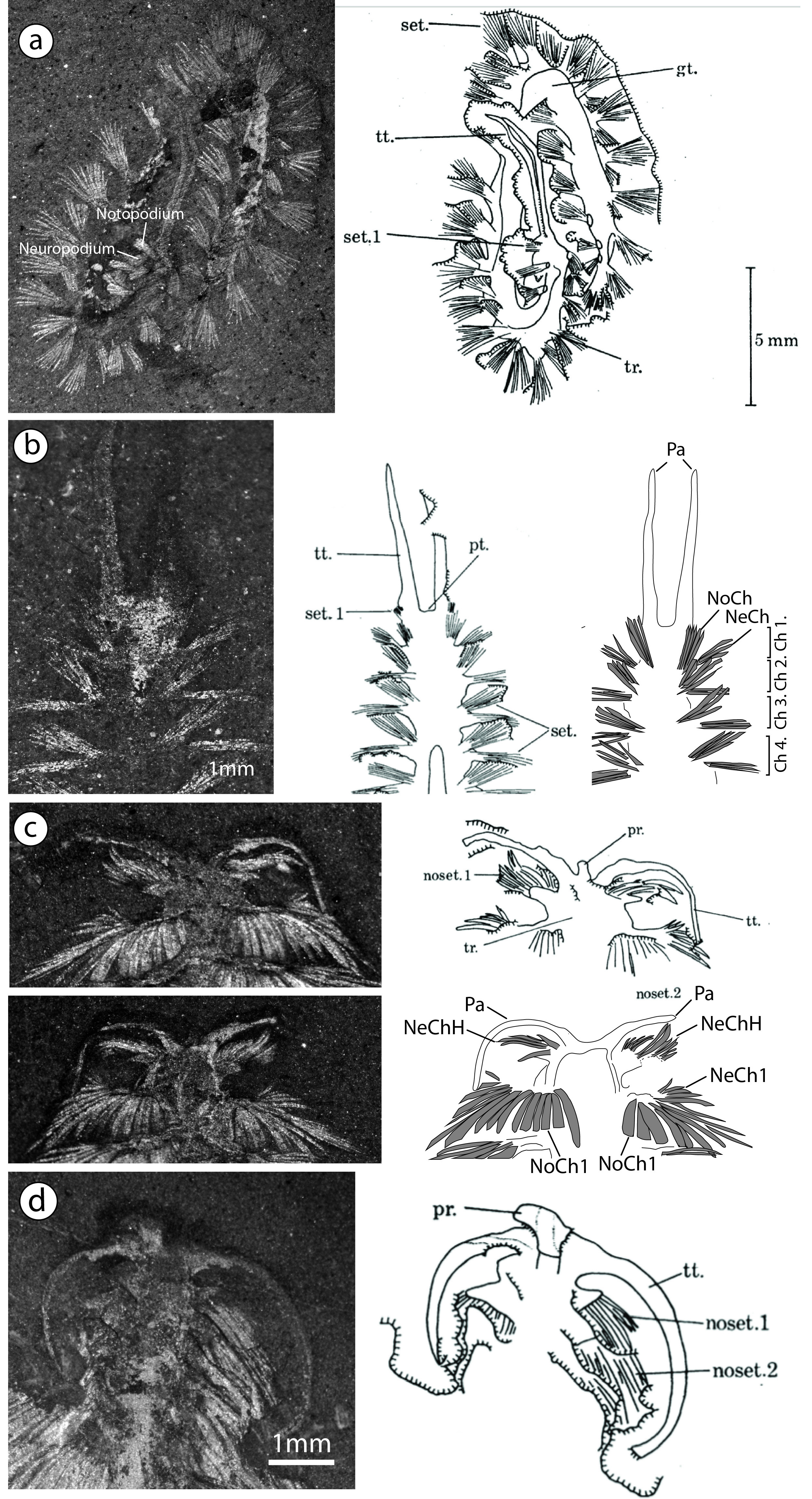

Supplement: Additional images of Burgess Shale polychaetes [file rsbl20150763supp1.jpg]

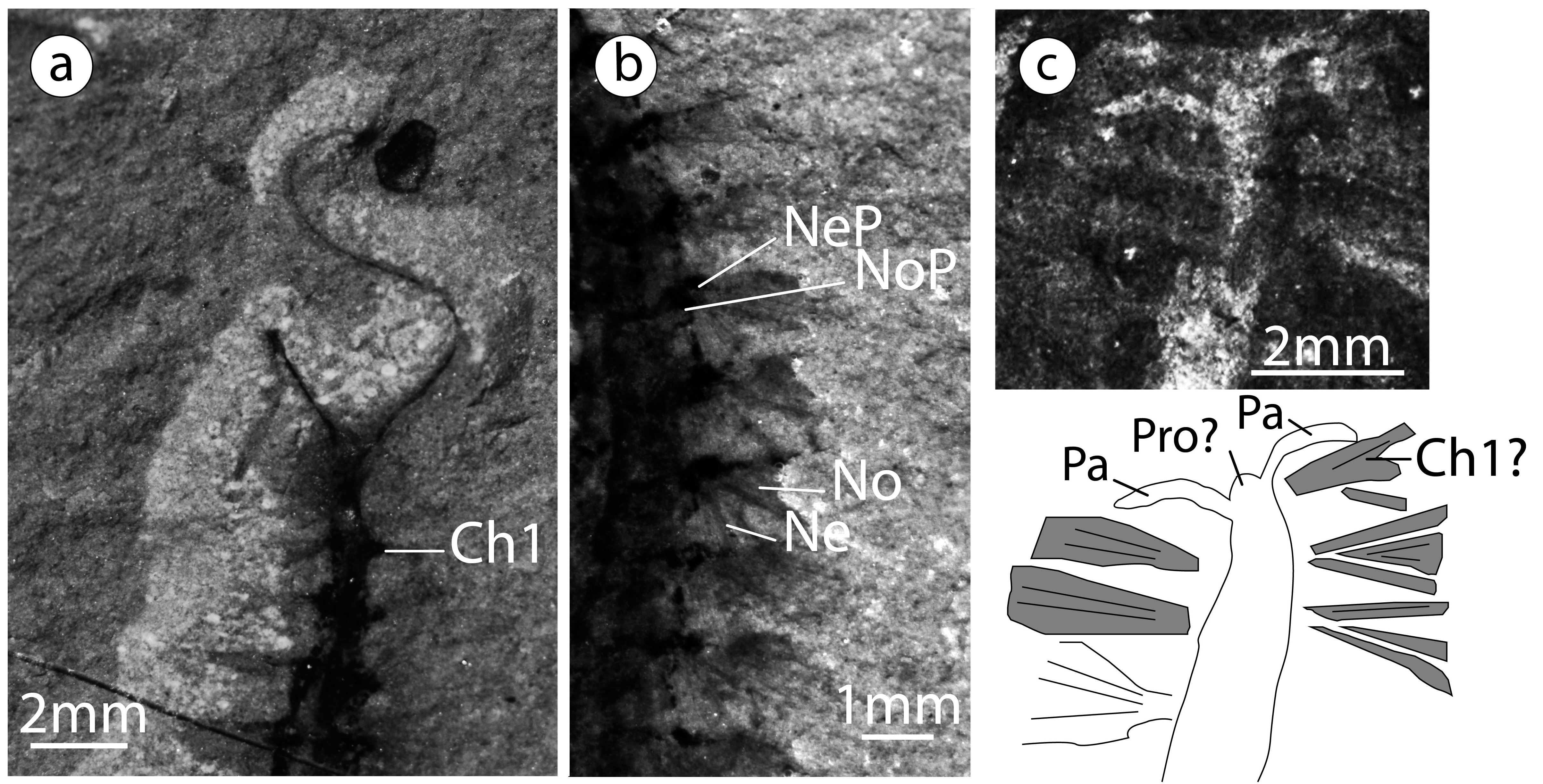

Supplement: Additional images of Marble Canyon and Sirius Passet polychaetes [file rsbl20150763supp2.jpg]
